# Natural Products for Cosmetic Applications

**DOI:** 10.3390/molecules28020534

**Published:** 2023-01-05

**Authors:** Jongsung Lee, Chang-Gu Hyun

**Affiliations:** 1 Department of Integrative Biotechnology, Sungkyunkwan University, Suwon 16419, Republic of Korea; 2 Jeju Inside Agency and Cosmetic Science Center, Department of Chemistry and Cosmetics, Jeju National University, Jeju 63243, Republic of Korea

Natural products provide an interesting and largely unexplored source for the development of potential new cosmetic ingredients. In some regions of the world, natural products are still the basis for the production of medicines and cosmetics. For many years, in highly developed countries, an increasing interest in medicinal and cosmetic products based on plant materials has been observed [1]. As a result, the global market demand for natural cosmetic ingredients such as plant extracts that can be used for depigmenting, anti-wrinkling, and other cosmeceutical uses is also increasing. Many companies around the world are trying to develop inhibitors or activators related to collagen synthesis, melanogenesis, and skin inflammation [2]. In addition, smart consumers using cosmetics tend to carefully review the mechanisms of action of these inhibitors or activators. Accordingly, this Special Issue (SI) on “Natural Products for Cosmetic Applications” is aimed at presenting novel data on natural products and single compounds with skin protection and improvement activities at either the enzymatic or cellular level. A number of original articles published in this issue examine (1) anti-hair loss, (2) inflammation, (3) skin aging including skin barrier strengthening and moisturizing, (4) melanogenesis, and (5) stretch marks and present the results of in vitro and in vivo studies. Finally, this SI, concerning innovative applications of natural products in the field of the cosmetic industry, contains 16 contributions, of which 14 are research articles and two are communications.

Some of these papers were inspired by information on the use of plants in traditional medicine, such as the photoaging effect of *Potentilla glabra* [3], wound healing and melanogenic inhibitory effect of *Angelica polymorpha* flower [4], stretch mark inhibition effect of *Lagerstroemia indica* flowers [5], anti-hair loss effect of *Ulmus davidiana* and *Connarus semidecandrus* [6,7], anti-aging effect of *Cirsium japonicum* flowers [8], and whitening effect of *Pouteria macrophylla* fruit [9].

Interestingly, new approaches such as biorenovation and drug repurposing were attempted in the development of skin health ingredients. Biorenovation, a microbial enzyme-assisted degradation process of precursor compounds, is an effective approach to unravel the potential bioactive properties of the derived compounds. Kim et al. and Park et al. produced luteolin-3′-O-phosphate and prunetin 4′-O-phosphate with bioconversion technology, inhibiting lipopolysaccharide-induced inflammatory responses by regulating NF-κB/MAPK cascade signaling in RAW 264.7 cells [10,11]. Kang et al. [12] attempted to uncover a new application of spiramycin, an old medication that was classically prescribed for toxoplasmosis and various other soft-tissue infections. They demonstrated that spiramycin can effectively attenuate the activation of macrophages, suggesting that spiramycin could be a potential candidate for drug repositioning as a topical anti-inflammatory agent. In addition, stretch marks are worth studying as raw materials for functional cosmetics. In the work of Yeom et al. [5], *Lagerstroemia indica* flower (LIFE) inhibited the adhesion of RBL-2H3 on fibronectin (FN) and the expression of integrin, a receptor for FN, thereby reducing focal adhesion kinase (FAK) phosphorylation. Ultimately, they demonstrated that LIFE suppresses FN-induced mast cell activation and promotes the synthesis of ECM components in fibroblasts, which indicates that LIFE may be a useful cosmetic agent for SD treatment.

In addition, keratinocytes and fibroblasts from anti-aging raw materials, papilla cells from bald patches, and mask cells for stretch marks were used as the cell types used in the development of functional materials for skin health.

In order to solve the hair loss problem, the authors set the following targets and conducted research: (1) transforming growth factor-beta, insulin-like growth factor 1, and dihydrotestosterone; (2) Wnt/β-catenin and autophagy; and (3) 5α-reductase activity and an intrinsic apoptotic pathway.

There are four main categories of signaling pathways, the activation of which during inflammation leads to the secretion of inflammatory mediators and pro-inflammatory cytokines: (1) I kappa B kinase (IκB)/nuclear factor kappa B (NF-κB), (2) mitogen-activated protein kinase (MAPK), (3) phosphoinositide 3-kinase, and (4) Janus kinase (JAK) signal transducer and activator of transcription (STAT) signaling pathways. In this SI, as targets for the development of anti-inflammatory materials, MAPK, IκB)/NF-κB, and STAT signaling pathways were applied.

Lastly, in the case of wrinkle improvement, research was conducted to inhibit ROS and apoptosis in keratinocytes, increase collagen production, and suppress MMPs in fibroblasts. In addition, the MAPK signaling pathway was focused on to strengthen the skin barrier and develop moisturizing ingredients.

To sum up, the SI “Natural Products for Cosmetic Applications” provides a current perspective of the natural products from marine and terrestrial areas and the rapidly developing research area, as evident from the resistance to the available drugs and the wide variety of chronic diseases. Considering the challenges in this exciting field of the discovery of natural product ingredients, this issue not only complements our knowledge on bioactive compounds, but may also uncover some novel ideas and motivation for the further investigation of various prospective biologically active compounds impacting human skin health practices. Finally, we wish to thank the invited authors for their interesting and insightful contributions and look forward to new advances in the bioactive ingredient field to be included in the following SI “Natural Products for Cosmetic Applications”.
